# 3,3'-Diindolylmethane induces gastric cancer cells death via STIM1 mediated store-operated calcium entry

**DOI:** 10.7150/ijbs.56833

**Published:** 2021-03-19

**Authors:** Yang Ye, Xue Li, Zhihua Wang, Fen Ye, Wenrong Xu, Rongzhu Lu, Haijun Shen, Shuhan Miao

**Affiliations:** 1 Department of Preventive Medicine and Public Health Laboratory Science, School of Medicine, Jiangsu University, Zhenjiang, China.; 2 Department of Gastroenterology, Affiliated Hospital of Jiangsu University, Jiangsu University, Zhenjiang, China.; 3 Department of Clinical Laboratory Center, Shaoxing People's Hospital (Shaoxing Hospital, Zhejiang University School of Medicine), Shaoxing, China.; 4 Key Laboratory of Medical Science and Laboratory Medicine of Jiangsu Province, School of Medicine, Jiangsu University, Zhenjiang, China.; 5 Center for Experimental Research, Affiliated Kunshan Hospital to Jiangsu University School of Medicine, Kunshan, Suzhou, China.; 6 Department of Health Care, Zhenjiang Fourth Peoples Hospital, Zhenjiang, China.

**Keywords:** 3,3'-Diindolylmethane, stromal interaction molecular 1, store-operated calcium entry, p-AMPK, endoplasmic reticulum stress, gastric cancer.

## Abstract

3,3'-Diindolylmethane (DIM), a natural phytochemicals isolated from cruciferous vegetables, has been reported to inhibit human gastric cancer cells proliferation and induce cells apoptosis as well as autophagy, but its mechanisms are still unclear. Store-operated calcium entry (SOCE) is a main Ca^2+^ influx pathway in various of cancers, which is activated by the depletion of endoplasmic reticulum (ER) Ca^2+^ store. Stromal interaction molecular 1 (STIM1) is the necessary component of SOCE. In this study, we focus on to examine the regulatory mechanism of SOCE on DIM-induced death in gastric cancer. After treating the human BGC-823 and SGC-7901 gastric cancer cells with DIM, cellular proliferation was determined by MTT, apoptosis and autophagy were detected by flow cytometry or Hoechst 33342 staining. The expression levels of related proteins were evaluated by Western blotting. Free cytosolilc Ca^2+^ level was assessed by fluorescence monitoring under a laser scanning confocal microscope. The data have shown that DIM could significantly inhibit proliferation and induce apoptosis as well as autophagy in two gastric cancer cell lines. After DIM treatment, the STIM1-mediated SOCE was activated by upregulating STIM1 and decreasing ER Ca^2+^ level. Knockdown STIM1 with siRNA or pharmacological inhibition of SOCE attenuated DIM induced apoptosis and autophagy by inhibiting p-AMPK mediated ER stress pathway. Our data highlighted that the potential of SOCE as a promising target for treating cancers. Developing effective and selective activators targeting STIM1-mediated SOCE pathway will facilitate better therapeutic sensitivity of phytochemicals acting on SOCE in gastric cancer. Moreover, more research should be performed to validate the efficacy of combination chemotherapy of anti-cancer drugs targeting SOCE for clinical application.

## Introduction

Gastric cancer is a malignant tumor of the digestive tract and its mortality rate second among all cancer worldwide^1^, Although diagnosis and treatment advanced recently, the five-year overall survival rate remains still low^1^.

3,3'-Diindolylmethane (DIM) is a natural phytochemical derived from cruciferous vegetables 2, our previous results have shown that it inhibited proliferation and induced apoptosis as well as autophagy in human liver cancer and gastric cancer 3-6. However, the underlying mechanism has not be fully elucidated.

The endoplasmic reticulum (ER) is a critical cellular organelle required for protein translation, modification, folding and assembly 7, 8. The accumulation of misfolded or unfolded proteins in the ER lumen could induce ER stress, followed by activation of the unfolded protein response (UPR) to restore ER homeostasis 9-11, The three unique sensors are associated with UPR: the PKR-like ER kinase (PERK), the inositol-requiring enzyme 1α (IRE1α) and the activation transcription factor 6 (ATF6) 12-14, however, if the cellular homeostasis fails to restore, cells death should occur 15. Transcription factor C/EBP homologous protein (CHOP) regulated by three UPR signaling pathway involved in ER stress-induced apoptosis and autophagy 16, 17. It was demonstrated that sesamin and quercetin could activate ER stress mediated apoptosis and autophagy in cervical cancer cells 18, 19. However, whether ER stress is responsible for DIM-induced cells death has not been determined.

The AMP-activated protein kinase (AMPK), mainly activated by Thr172 phosphorylation in response to metabolic stress, is a crucial cellular energy sensor 20-22, its phosphorylation targets induces the phosphorylation of Acetyl-CoA Carboxylase (ACC) 23, 24. It have been shown that the activation of AMPK plays important role in cell survival and apoptosis 25. Chicoric acid phosphorylated p-AMPK to activate ER stress and subsequent autophagy 26. However, the role of p-AMPK/p-ACC in anti-cancer effect of DIM remains to be established.

Ca^2+^ is a common intracellular second messenger and regulate several cancer processes such as proliferation, migration 5. The increase of cytosolic Ca^2+^ could activate Calpain, Ca^2+^-dependent cysteine protease to regulate a series of signaling pathway 34. It has been shown that the high level of cytosolic Ca^2+^ was related to the apoptosis 16, Abdoul-azize et al reported that simvastation induced breast cancer cells apoptosis mainly via disturbing Ca^2+^ homeostasis 27. Store-operated calcium entry (SOCE) is the major Ca^2+^ influx pathway in non-excitable cells, which occurring following decrease in the Ca^2+^ level of ER lumen, stromal interaction molecular 1 (STIM1) on the ER membrane is the necessary component of SOCE 28. Accumulating studies indicated that dysregulation of SOCE has implicated in various of cancer cells tumorigenesis and progression 29. Cisplatin mainly targets to activate SOCE to induce non-small cell lung cancer cells apoptosis 30. In addition, Chiu has reported that SOCE mediate apoptosis through the activation of ER stress 31. SOCE could also regulate human neuroblastoma energy metabolism via activating p-AMPK 32. Therefore, exploring the role of SOCE in DIM-induced cells apoptosis and autophagy may be beneficial to improving of therapeutic effect in cancer treatment.

In the present study, we aim to investigate the regulatory mechanism of STIM1 mediated SOCE in DIM-induced cell autophagy and apoptosis and explore the role of AMPK signaling pathway and ER stress in anticancer effects of DIM.

## Materials and methods

### Chemicals and reagents

3,3'-Diindolylmethane (DIM) (catalog no. BML-GR207), p-AMPK inhibitor Compound C, intracellular Ca^2+^ chelator BAPTA-AM, SOCE nonspecific inhibitor 2-APB, Fluo3/AM obtained from Sigma-Aldrich (St. Louis, MO, USA), Mag-Fluo-4/AM (M14206) and lipofectamin 2000 were purchased from invitrogen (Carlsbad, CA, USA). DIM was dissolved in dimethylsulfoxide (DMSO) and prepared into 100mM concentration stock solution, which was stored at 4 °C. 3-[4,5-dimethylthizaol-2-yl]-2,5-diphenyl-2H-tetrazolium bromide (MTT), Hoechst 33342, paraformaldehyde (PFA), bicinchoninic acid (BCA) protein assay kit (P0011) were purchased from beyondtime (Shanghai, China). The flow cytometry reagent was purchased from BD bioscience (San Jose, CA, USA). Primary antibodies against STIM1 (1:1000, abcam), phosptho-AMPK (1:1000, CST), phosptho-ACC (1:1000, CST), Calpain (1:1000, immunoway), phosptho-PERK (1:1000, CST), ATF6 (1:1000, CST), CHOP (1:1000, CST), phosptho-IRE1α (1:1000, abcam), Bcl-2 (1:1000, CST), Bax (1:1000, CST), cleaved-caspase3 (1:1000, CST), LC3B (1:1000, CST), primary antibodies against GAPDH (1:10000) and horseradish peroxidase (HRP)-labeled secondary antibodies were purchased from Santa Cruz Biotechnology (Santa Cruz, CA, USA).

### Cell culture and drug treatment

The human BGC-823 and SGC-7901 gastric cancer cell lines were purchased from the Shanghai Institute of Biochemistry and Cell Biology, Chinese Academy of Sciences (Shanghai, China), cells were cultured in RPMI 1640 medium (Gibco Life Technologies, Grand Island, NY, USA) containing 10% fetal bovine serum (Tianhang Biological Technology Co, Ltd, Hangzhou, Zhejiang, China) at 37 °C in a 5% CO_2_ humidified atmosphere. Cells were treated continuously with DIM at a concentration ranging from 0-120μM for varying periods of time ranging from 24h to 48h. p-AMPK inhibitor Compound C (10μM), Ca^2+^ chelator BAPTA-AM (7.5μM), SOCE non-specific inhibitor 2-APB (20μM) was used to pre-treat BGC-823 gastric cancer cells for 30min prior to DIM exposure.

### MTT assay

BGC-823 and SGC-7901 gastric cancer cells (5000 cells/well) were seeded in 96-well plate and then exposed to DIM at various concentrations and time. Then 10μL MTT solution (0.5mg/mL) was supplemented to incubate for 3-4h at 37 °C. Finally, the medium was discarded and 150μL DMSO of each well added. Cell viability was measured using the microplate reader as absorbance at 490nm. The following formula was employed to calculate relative cell viability rate (%), i.e. (OD value of experimental group/OD value of control group) × 100 %.

### Hoechst 33342 staining

After 24 hours of treatment with various of DIM concentrations (0, 10, 20, 40, 60 and 80μM), cells were washed three time with PBS and fixed with 4% PFA, following staining with Hoechst 33342 for 20min, and washed the three times with PBS. Cells were then covered with PBS and observed in common fluorescence microsope.

### Apoptosis detection by flow cytometry analysis

The BGC-823 and SGC-7901 gastric cancer cells (5×10^5^/well) were seeded in 6-well plate and overnight incubation at 37 °C, the cells were treated with the different concentrations (0, 10, 20, 40, 60 and 80μM) of DIM for 24h. Following harvested with trypsin and washed three times with PBS. Next the cells were stained with 5μL FITC conjugated Annexin V and 5μL propidium (PI) in 500μL binding buffer at room temperature for 20min in the dark. Finally the apoptotic cells were quantified using a flow cytometry (BD bioscience USA).

### Small interfering RNA transfection

BGC-823 gastric cancer cells were transfected with 50nM CHOP siRNA, STIM1 siRNA or negative control siRNA using lipofectamine 2000 reagent according to the manufacturer's instruction respectively, 48h after transfection, cells were treated with either 80μM DIM or 0.1% DMSO for another 24h, followed by the MTT assay or Western blotting. The siRNA sequence as following: the human CHOP-siRNA: 5'-GGCTCAAGCAGGAAATCGA-3'. The human STIM1-siRNA: 5'-GTGGTACAGTGGCTGATCA-3'.

### Protein extraction and immunoblotting

Cells were washed with ice-cold PBS and homogenised with lysis buffer. After 30min of incubation on ice, whole-cell extracts were pelleted in an Eppendorf microcentrifuge at 12000rpm for 15min at 4 °C, then stored at -20 °C or immediately subjected to sodium dodecyl sulfate polyacrylamide gel electrophoresis. The protein concentration was measured with the BCA method. A total of 40μg protein were used for immunnoblotting analysis then incubated with primary antibodies against the mentioned proteins at 4 °C overnight. The membranes were incubated with the corresponding horseradish peroxidase-conjugated secondary antibody for 1h.

### Ca^2+^ measurement

Ca^2+^ measurement was performed on attached populations of BGC-823 and SGC-7901 gastric cancer cells with DIM for 24h. The treated cells were immersed with 5μM Fluo-3/AM or Mag-Fluo-4/AM for 60 min at 37 °C in RPMI 1640 medium then was washed three times with PBS at 37 °C. After that, all cells were incubated with Hoechst 33342 staining for 20min and washed three times with PBS. Fluorescence was monitored under a Laser Scanning Confocal Microscope.

### Statistical analysis

Experiments were performed at least three times with similar results. The results were presented at the mean±standard deviation (SD) after the analyses were completed with Graph-Pad Prism 6.0 (Graph Pad Software, San Diego, CA, USA). One-way analysis of variance was used to assessthe significance of differences among groups, when the value of *p<0.05 was considered significant.

## Results

### 3,3'-Diindolemethane (DIM) inhibits proliferation and induces apoptosis as well as autophagy in BGC-823 and SGC-7901 gastric cancer cell lines

**Figure 1:**
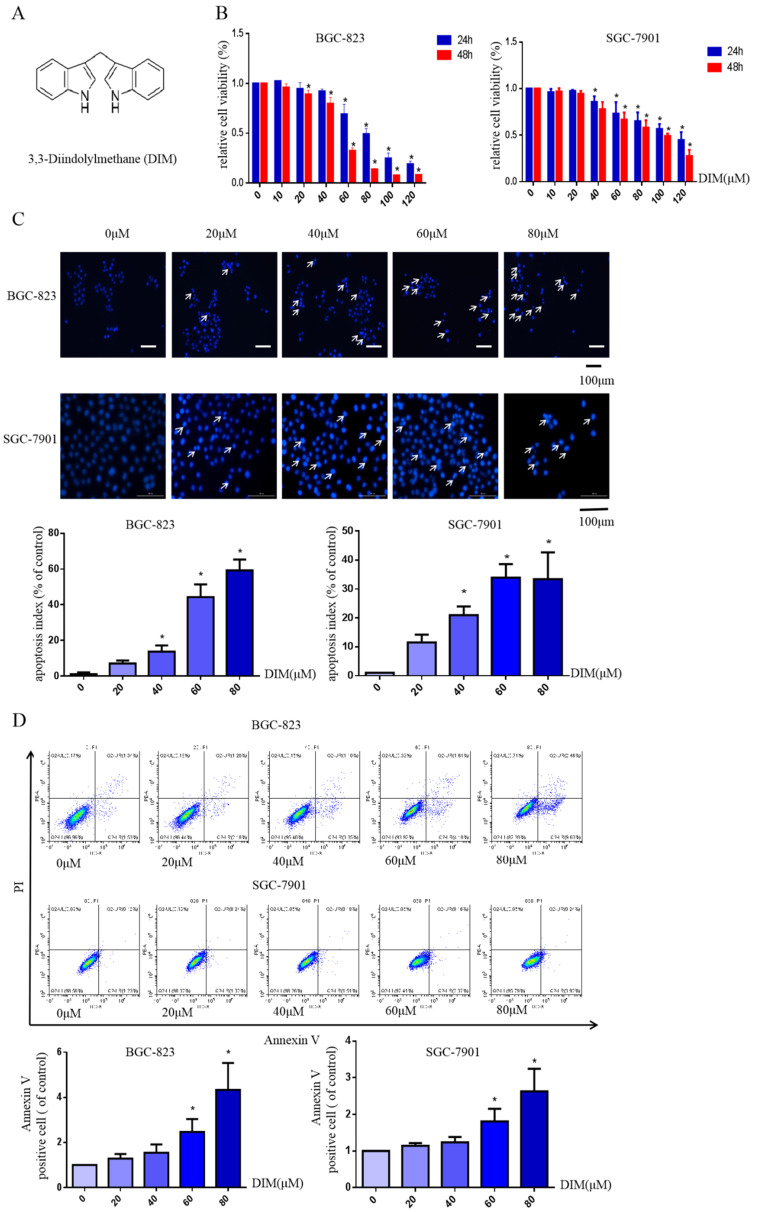

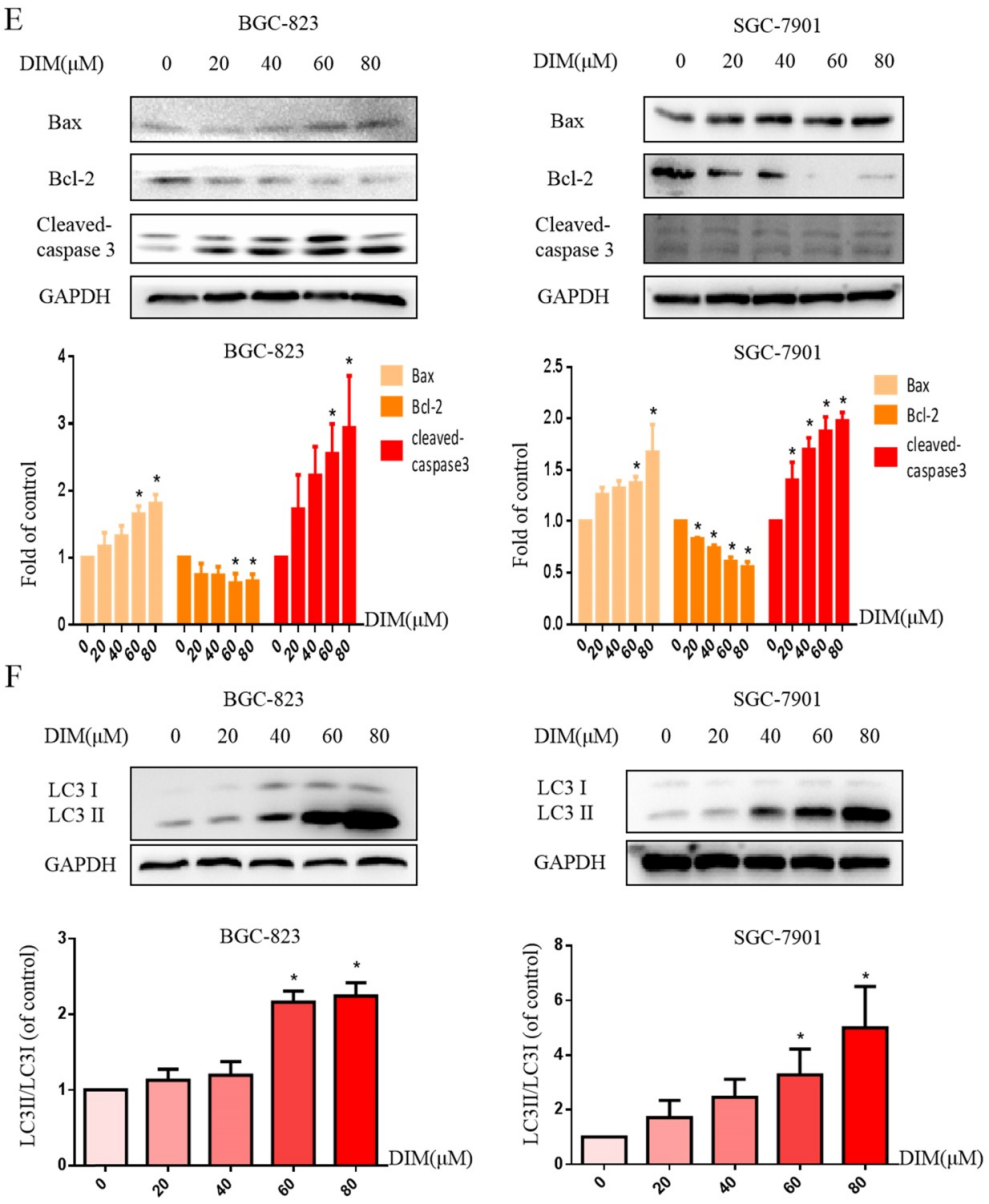
** DIM reduces cell viability and induces autophagy and apoptosis in gastric cancer.** (A) The structural component of DIM. (B) Cells were treated with different DIM (0-120μM) for 24, 48h, which viability was determined by MTT assay. The results are presented as mean±SD and described as column chart *p<0.05 as compared with control group. (C) Hoechst 33342 staining was used to evaluate apoptosis levels with treatment of DIM for 24h. (D) Flow cytometry was assessed cells apoptosis with DIM treatment. (E) Cells apoptotic proteins analysis of DIM treated cells. BGC-823 and SGC-7901 gastric cancer cells treated with DIM (0-80μM) for 24h. The expression of cell key apoptosis-related protein was detected by Western-blotting. DIM significantly increase the pro-apoptotic marker Bax, cleaved-caspase 3 and decrease anti-apoptotic marker Bcl-2 compared control group. (F) Cells exposure with DIM (0-80μM) for 24h, the expression of autophagy-related proteins was detected by Western-blotting. DIM significantly increase LC3II/LC3I. Values represent as the mean±SD of three independent experiments (n=3) (*p<0.05 compared with the control group).

The structural formula of DIM was shown in Figure 1A. The human BGC-823 and SGC-7901 gastric cancer cells were treated with concentrations of DIM (0-120μM) for 24h or 48h, then results of MTT assay indicated that DIM decreased cells viability in a concentration manner (Figure 1B).

Hoechst 33342 staining and flow cytometry analysis demonstrated that DIM significantly increased the rate of apoptotic cells in a concentration dependent manner (Figure 1C, D), in addition, DIM exposure for 24h significantly decreased Bcl-2 and increased Bax, cleaved-caspase 3, three important indicator of apoptotic pathway (Figure 1E). Also DIM significantly induced autophagy as evidenced by increasing percentages of endogenous LC3-II conversion in a concentration-dependent manner (Figure 1F). These results revealed that DIM inhibit BGC-823 and SGC-7901 gastric cancer cells proliferation and promote apoptosis as well as autophagy. Subsequently, we mainly used BGC-823 cancer cell line as the experimental object to explore the specific mechanism of anti-cancer effect of DIM.

### DIM inhibits cells growth through CHOP-related ER stress in BGC-823 gastric cancer cells

**Figure 2:**
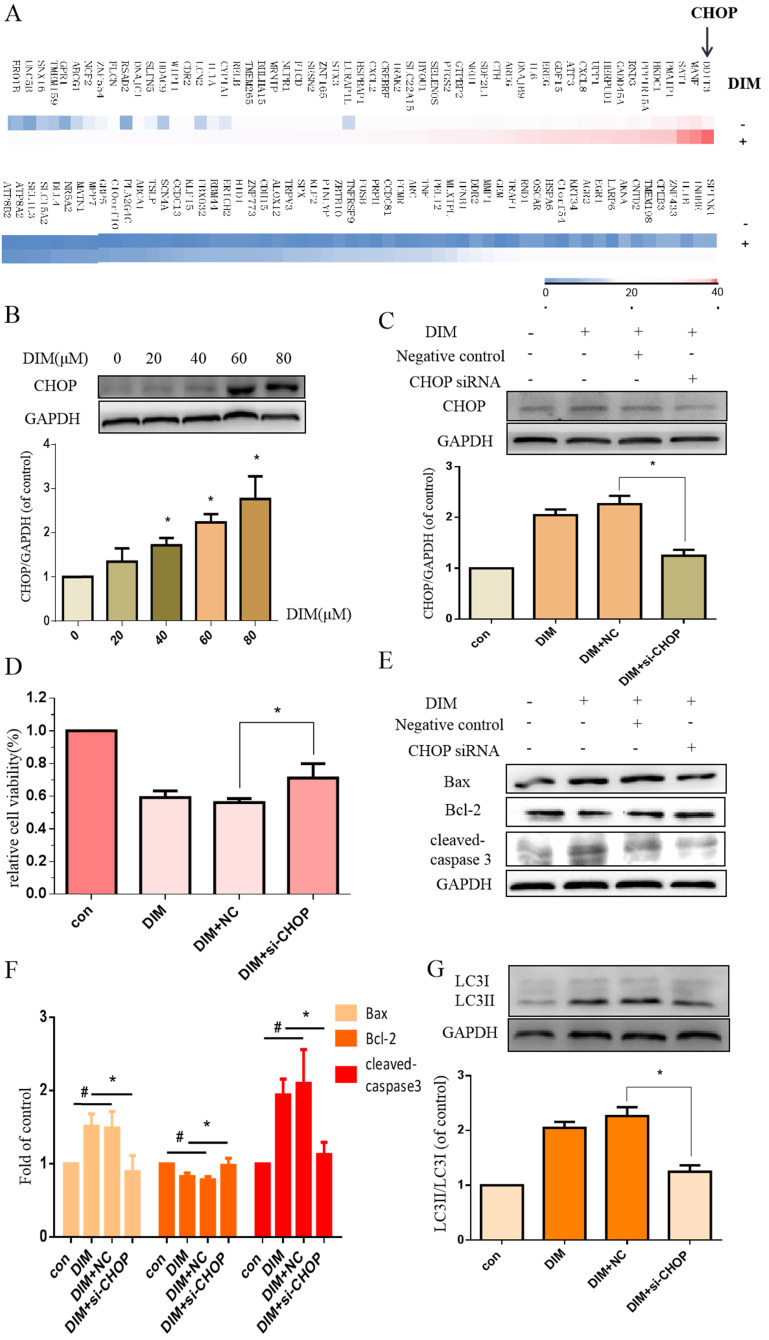
** DIM induces endoplasmic reticulum (ER) stress in BGC-823 gastric cancer cells.** (A) The CHOP mRNA was significantly higher in the DIM treatment group compared to untreated group through High-throughput sequencing. (B) Cells were treated with different dose of DIM (0, 20, 40, 60, 80μM) for 24h, the levels of C/EBP homologous protein (CHOP) was detected by Western blot. (C) The efficiency of CHOP knockdown was detected by Western blot in cells transfected with negative control siRNA or CHOP siRNA. (D) Cells were transfected with control siRNA or CHOP siRNA for 48h, followed by incubation with 80μM DIM for another 24h, cell viability was measured by the MTT assay. The results are presented as mean±SD and described as column chart *p<0.05 as compared with control group. (E, G) The protein levels of Bax, Bcl-2, cleaved-caspase 3, LC3II/LC3I was detected by Western blot in cells transfected with NC siRNA or CHOP siRNA. (F) Quantitative analysis of protein levels. (Values represent as the mean±SD of three independent experiments (n=3) (*p<0.05 compared with the control group)).

To investigate whether DIM-induced cell death is associated with ER stress, we firstly performed RNA sequencing analysis of cells treated with DIM or DMSO for 24h to identify specific signaling pathway, the data showed that the levels of CHOP mRNA were prominently increased in DIM treated cells compared to control group (Figure 2A), subsequently, DIM significantly increased expression of CHOP protein in a concentration dependent manner (Figure 2B), indicated that the occurrence of ER stress in DIM induced cells death. To further explore the role of ER stress in DIM induced cell autophagy and apoptosis, CHOP siRNA was transfected into BGC-823 gastric cancer cells. The knockdown efficiency as shown in Fig. 2C. Cells viability was reversed in DIM concomitant with CHOP siRNA compared to the group of co-treatment DIM with negative control siRNA (Figure 2D), moreover, the knockdown of CHOP attenuated DIM induced Bax, cleaved-caspase 3, LC3II/LC3I and decrease of Bcl-2 (Figure 2E-G). These results suggest that DIM-induced apoptosis and autophagy is partially mediated by the CHOP-related ER stress pathway in BGC-823 gastric cancer cells.

### DIM up-regulate CHOP through ATF6 in BGC-823 gastric cancer cells

**Figure 3:**
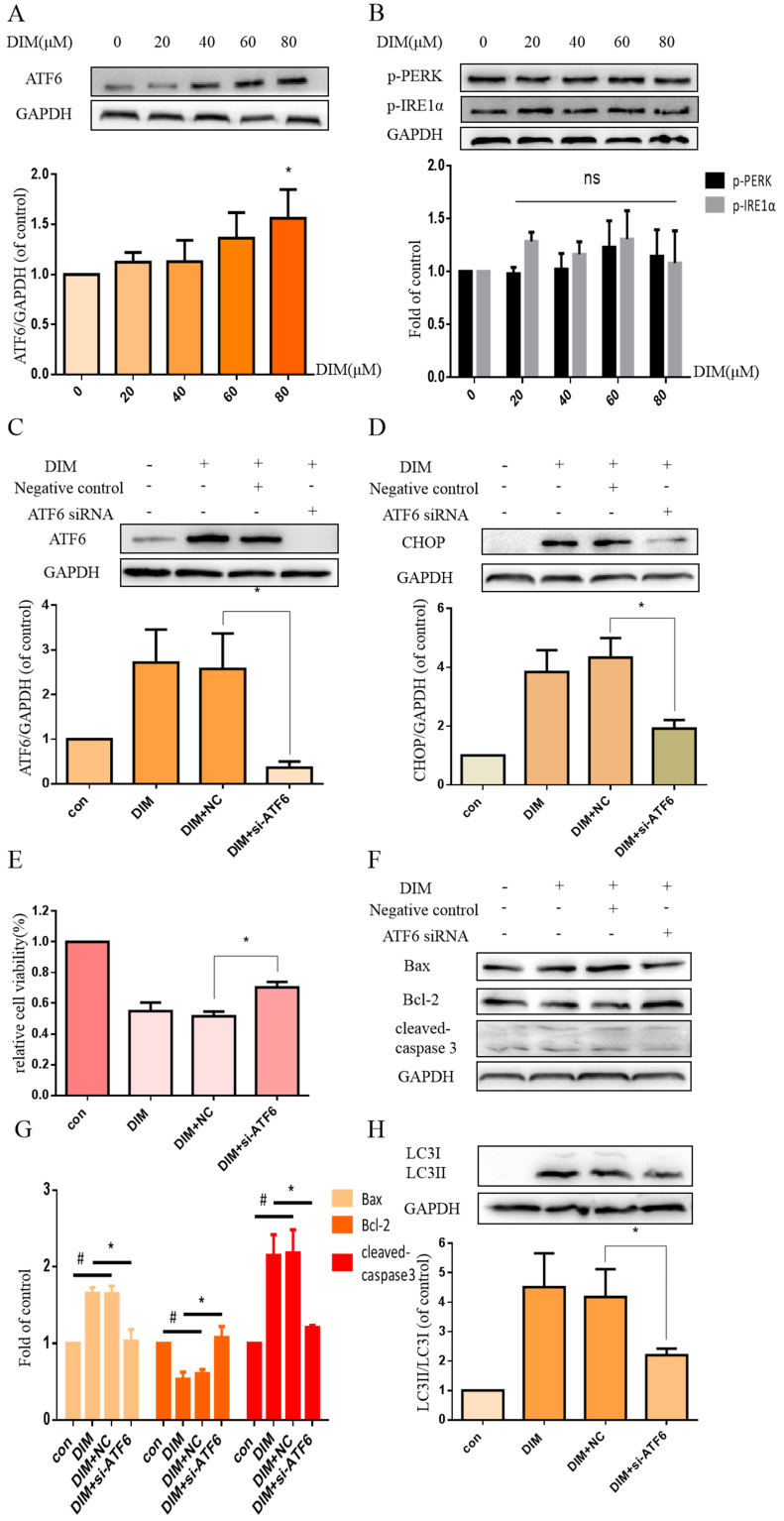
** DIM triggers the activation of UPR.** (A, B) BGC-823 gastric cancer cells were treated with 80μM DIM for 24h. The levels of p-PERK, ATF6, p-IRE1α were analyzed by western blot. Quantitative analysis of protein levels. Values are expressed as the mean±SD of three independent experiments (n=3) (*p<0.05 compared with the control group). (C) The knockdown efficiency of ATF6 was evaluated by western blot in cells transfected with negative control siRNA or ATF6 siRNA. (D, F, H). The protein levels of CHOP, Bax, Bcl-2, cleaved-caspase 3, LC3II/LC3I was detected by Western blot in cells transfected with NC siRNA or CHOP siRNA. (E) Cells were transfected with NV siRNA or ATF6 siRNA for 48h, followed by incubation with 80μM DIM for another 24h, cells viability was measured by the MTT assay. The results are presented as mean±SD and described as column chart *p<0.05 as compared with control group. (G) Quantitative analysis of protein levels. (Values represent as the mean±SD of three independent experiments (n=3)(*p<0.05 compared with the control group)).

To further investigated whether DIM activated the UPR to induce ER stress, cells were treated with DIM for 24h and the expression of p-PERK, p-IRE1α, ATF6 analyzed by western blot, the result showed that ATF6 was clearly activated by DIM in a concentration dependent manner (Figure 3A), but IRE1α and PERK phosphorylation was not change (Figure 3B). Subsequently, ATF6 siRNA was transfected into cells, the knockdown efficiency as shown in figure 3C. The knockdown of ATF6 could significantly reversed DIM-induced CHOP (Figure 3D). Moreover, cells viability was attenuated in DIM combined with ATF6 siRNA compared to the group of co-treatment DIM with negative control siRNA (Figure 2E), DIM induced Bax, cleaved-caspase 3, LC3II/LC3I and decrease of Bcl-2 also attenuated (Figure 2F-H). These demonstrated that DIM mainly induce CHOP by upregulating ATF6.

### DIM enhanced p-AMPK to induce ER stress and cells death in BGC-823 gastric cancer cells

**Figure 4:**
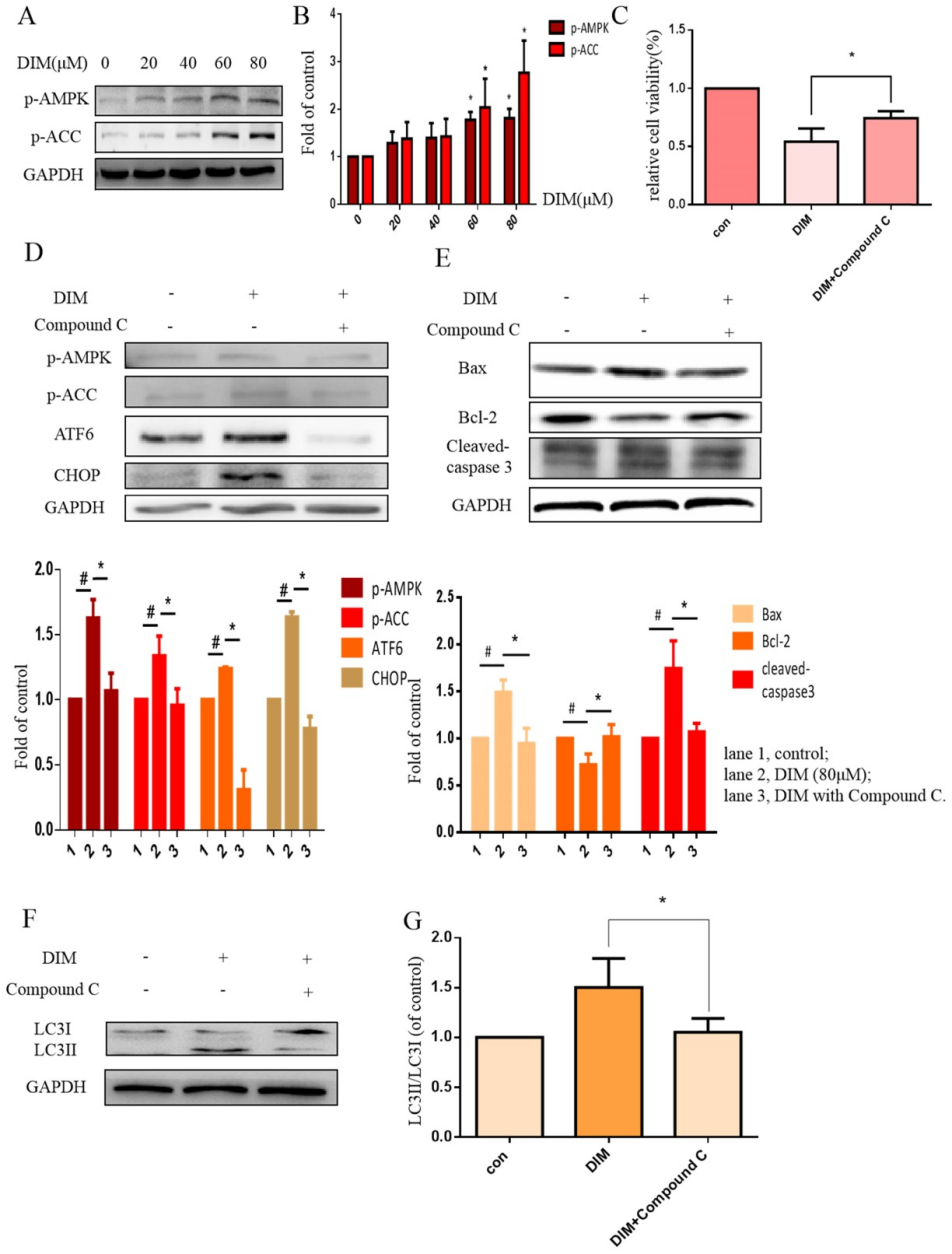
**AMPK signaling activating ER stress was involved in DIM-induced cells death.** (A) Effect of DIM on AMPK/ACC signaling pathway, cells were treated with DIM (0μM, 20μM, 40μM, 60μM, 80μM) for 24h. The levels of protein was evaluated by Western blot. Representative of three independent experiments was shown in (B). (C) Cells were treated with DIM (80μM) with or without Compound C (10μM) for 24h, after which cell viability was measured using the MTT assay. (D) The protein levels of p-AMPK, p-ACC, ATF6, CHOP was detected after treating with DIM (80μM), Compound C (10μM) for 24h. (E, F) The Bax, Bcl-2, cleaved-caspase 3, LC3II/LC3I was evaluated by Western blot after treatment DIM and Compound C. (G) Quantitative analysis of protein levels. (The data represent mean± SD of three independent experiments (n=3) (*p<0.05 compared with the control group, #p<0.05 compared with the DIM group)).

Phosphorylation of AMPK has been reported to mediate ER stress 32. Our work found that DIM phophorylated AMPK and ACC in a concentration dependent manner (Figure 4A, B). Furthermore, to evaluate the role of p-AMPK/p-ACC in ER stress mediated cells apoptosis and autophagy by DIM, Compound C, a p-AMPK inhibitor, was used, MTT assay showed that the anti-cancer effect of DIM was attenuated (Figure 4C). Co-treatment of DIM and Compound C significantly reversed DIM-induced ATF6, CHOP (Figure 4D). Moreover, effects of DIM on the expression of Bax, Bcl-2, cleaved-caspase 3 and LC3II/LC3I were also partially attenuated (Figure 4E-G). These results suggest that DIM-induced apoptosis and autophagy may be regulated through p-AMPK mediated ER stress.

### DIM increases cytoplasmic free Ca^2+^ level

**Figure 5:**
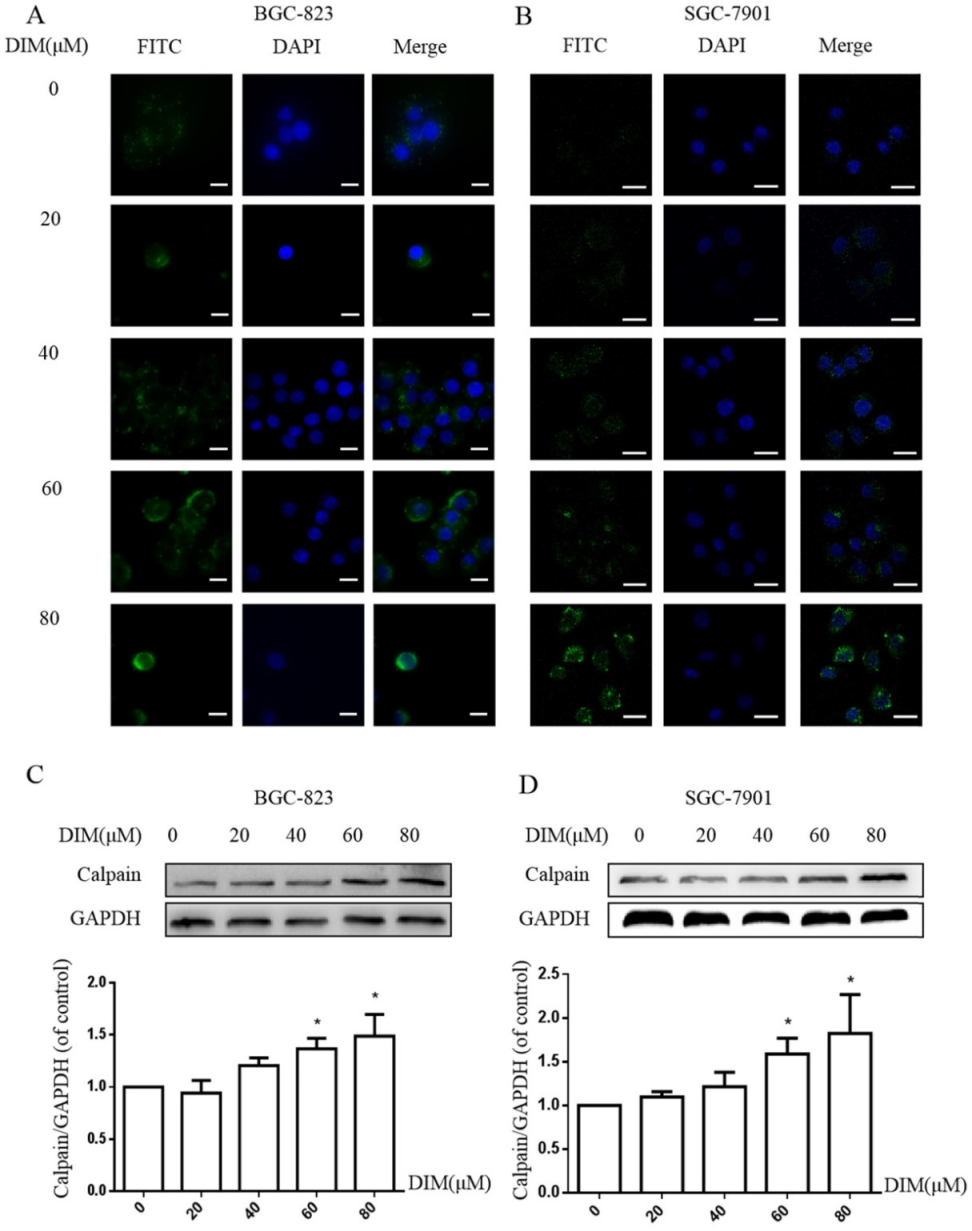

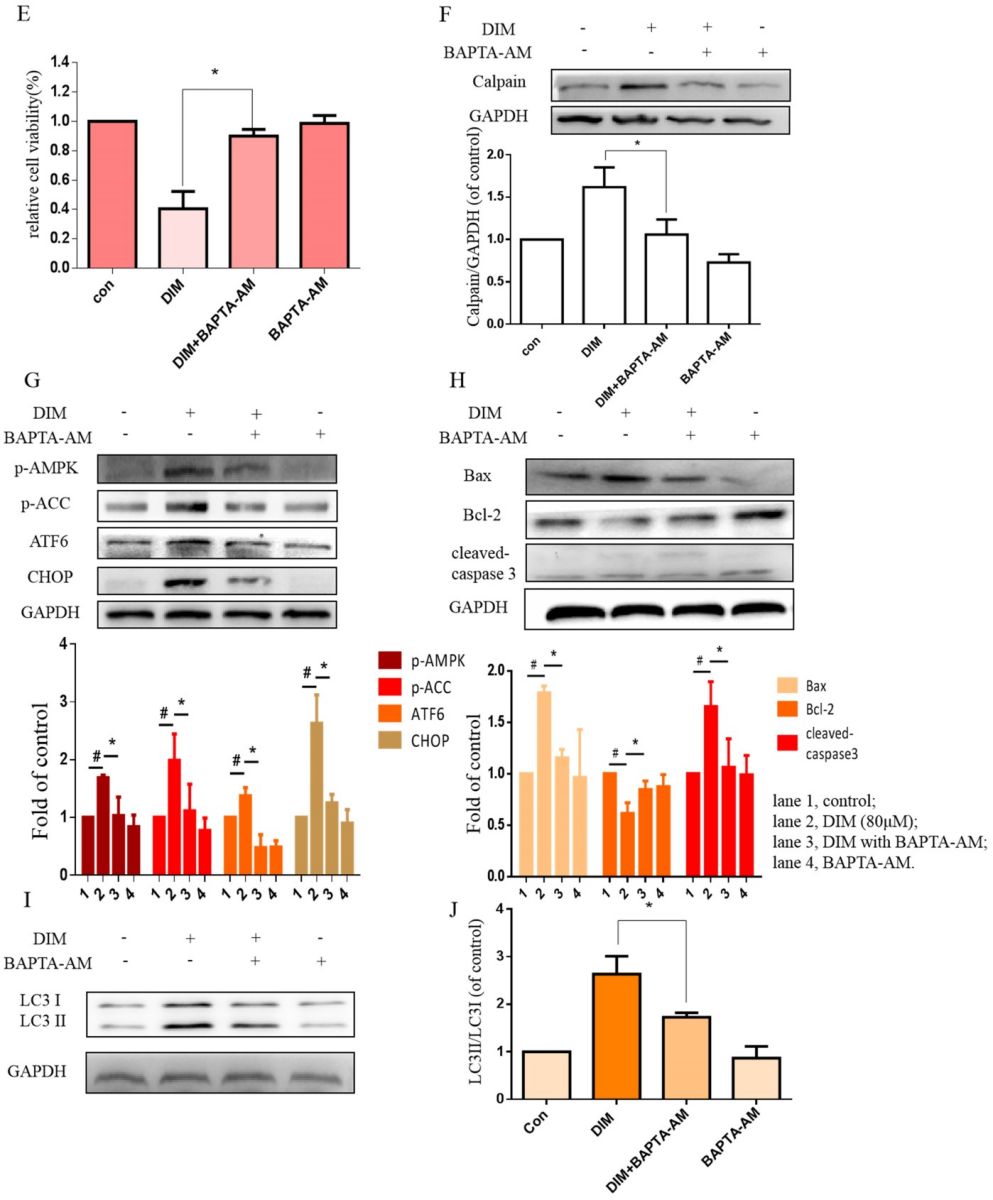
** Cytoplasmic free calcium was involved in DIM-induced cells death by p-AMPK-mediated ER stress.** (A) The human BGC-823 gastric cancer cells were treated with different concentration of DIM for 24h, fluorescence intensity of Ca^2+^ was measured by laser scanning confocal microscope. Scale bar: 15μm. (B) Fluorescence intensity of Ca^2+^ was measured by laser scanning confocal microscope in SGC-7901 gastric cancer cells. Scale bar: 25μm. (C, D) Western blot analysis of the levels of Calpain following with DIM (0μM, 20μM, 40μM, 60μM, 80μM) for 24h in BGC-823 and SGC-7901 gastric cancer cells. (E) Cells were treated with DIM (80μM), BAPTA-AM (10μM) for 24h, cell viability was detected by MTT assay in BGC-823 gastric cancer cells. (F, G, H, I). The expression of Calpain, p-AMPK, p-ACC, ATF6, CHOP, Bax, Bcl-2, cleaved-caspase 3, LC3II/LC3I were detected by Western blot after treatment of DIM with BAPTA-AM. (J) Quantitative analysis of protein levels. (The data represent mean± SD of three independent experiments (n=3) (*p<0.05 compared with the control group, #p<0.05 compared with the DIM group)).

The activation of STIM1-mediated SOCE could increase sustained cytoplasmic free Ca^2+^ overload 31, the treatment of DIM for 24h enhanced the Ca^2+^ fluorescence intensity by Laser scanning confocal microscope in BGC-823 and SGC-7901 gastric cancer cells (Figure 5A, B). Furthermore, the levels of Calpain significantly increased after the treatment with DIM (Figure 5C, D), since cytoplasmic Ca^2+^ elevation could activate Calpain 33. These results indicated that DIM leaded to cytoplasmic Ca^2+^ overload in gastric cancer.

To further determine effects of cytoplasmic Ca^2+^ on DIM-induced growth inhibition, cytoplasmic Ca^2+^ chelator, BAPTA-AM was used in BGC-823 gastric cancer cells, cells growth inhibition and Calpain by DIM was partially attenuated (Figure 5E, F). Moreover, compared to the DIM-only treatment group, the expression of p-AMPK, p-ACC, ATF6, CHOP was also decreased with BAPTA-AM and DIM combination treatment (Figure 5G). These indicated that cytoplasmic Ca^2+^ is involved in DIM-induced AMPK mediated ER stress signaling pathway. In addition, BAPTA-AM also significantly blocked the upregulation of Bax, cleaved-caspase 3, LC3II/LC3I and decrease of Bcl-2 by DIM (Figure 5H-J). These findings demonstrated that the DIM-induced apoptosis and autophagy was associated with the cytoplasmic Ca^2+^ overload.

### DIM activate STIM-mediated SOCE in BGC-823 and SGC-7901 gastric cancer cells

**Figure 6:**
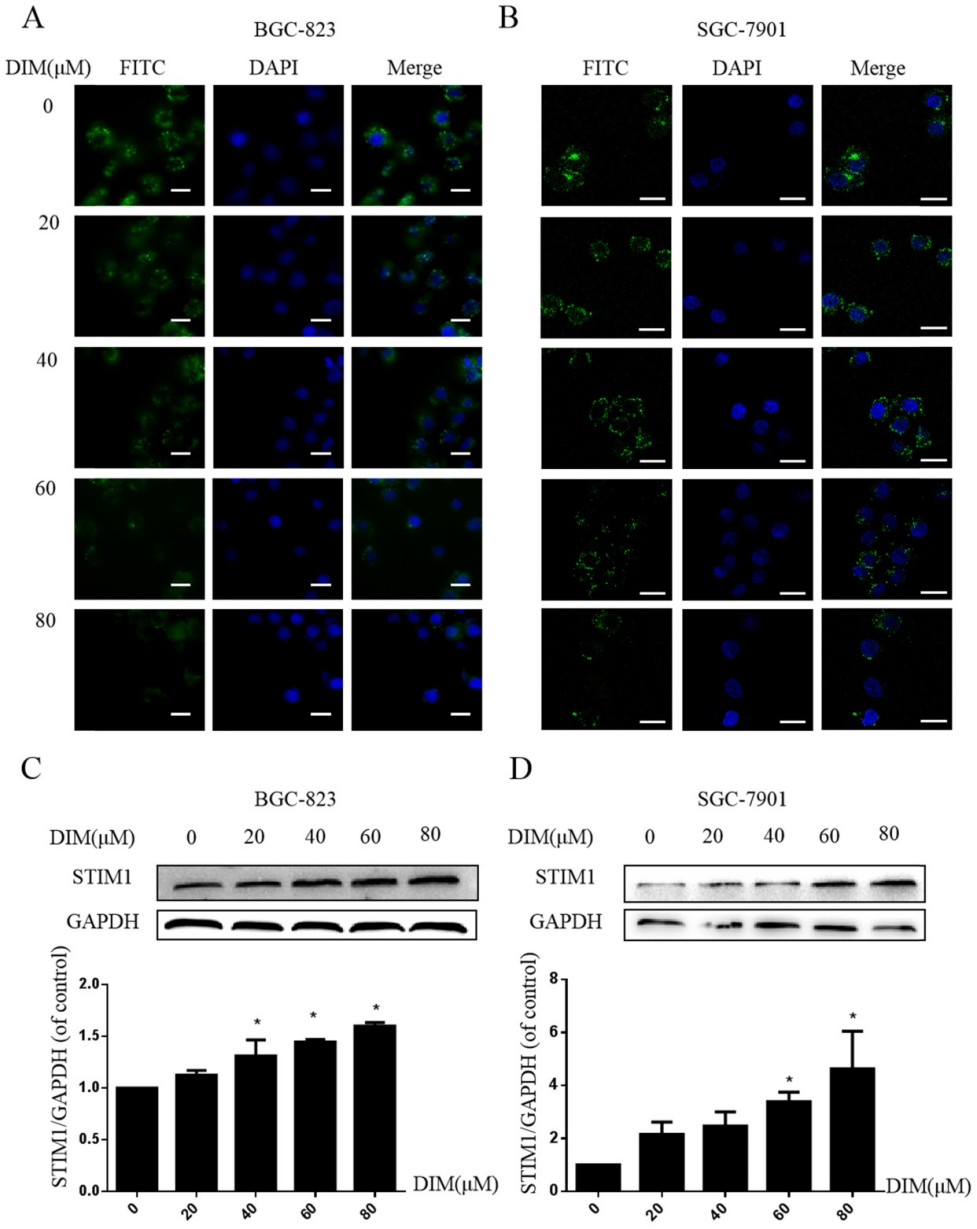

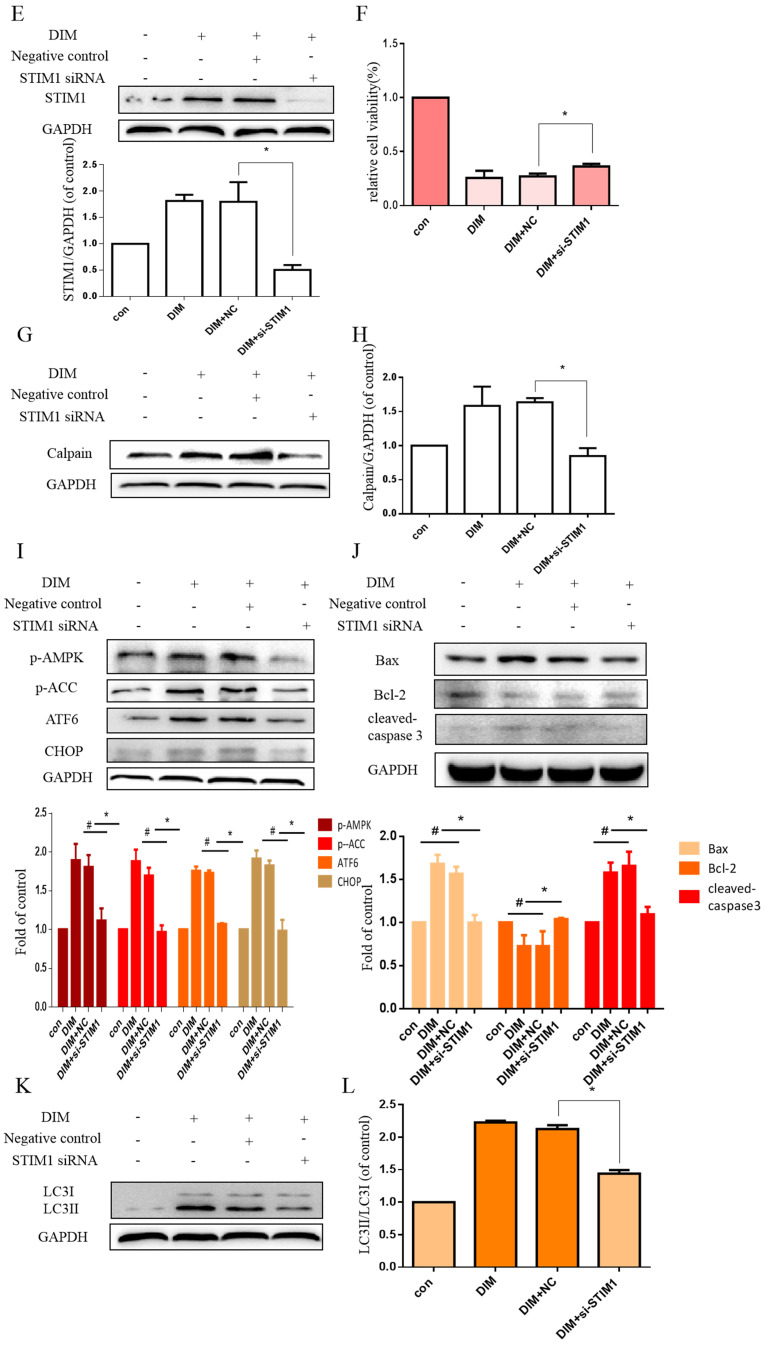
** Effect of SOCE on DIM-induced cell apoptosis and autophagy by p-AMPK-mediated ER stress in BGC-823 gastric cancer cells.** (A) Cells were treated with different DIM for 24h, fluorescence intensity Ca^2+^ in ER store was measured by laser scanning confocal microscope. Scale bar: 15μm. (B) fluorescence intensity Ca^2+^ in ER store was measured by laser scanning confocal microscope in SGC-7901. Scale bar: 25μm. (C, D) Western blot analysis of the levels of STIM1 after treatment with different DIM (0μM, 20μM, 40μM, 60μM, 80μM) for 24h. (E) The knockdown efficiency of STIM1 was detected by Western blot in BGC-823 gastric cancer cells transfected with NC siRNA or STIM1 siRNA. (F) Cells were transfected with NC siRNA or STIM1 siRNA for 48h, followed by incubation with 80μM DIM for another 24h, cells viability was detected by the MTT assay. (G, H, I, J, K, L) The protein level of Calpain, p-AMPK, p-ACC, ATF6, CHOP, Bax, Bcl-2, cleaved-caspase 3, LC3II/LC3I was detected by Western blot in cells transfected with control siRNA or STIM1 siRNA. (Values represent as the mean±SD of three independent experiments (n=3)(*p<0.05 compared with the control group, #p<0.05 compared with the DIM group)).

Cells were treated with DIM for 24h and loaded with Ca^2+^-sensitive fluorescent dye Mag-Fluo-4/AM, then ER store Ca^2+^ fluorescence intensity was measured by Laser scanning confocal microscope, DIM apparently decreased the ER Ca^2+^ level (Figure 6A, B). In addition, expression of STIM1 was upregulated by DIM in a dose-dependent manner (Figure 6C, D). These data initially suggested that DIM could activate STIM1-mediated SOCE.

**Figure 7:**
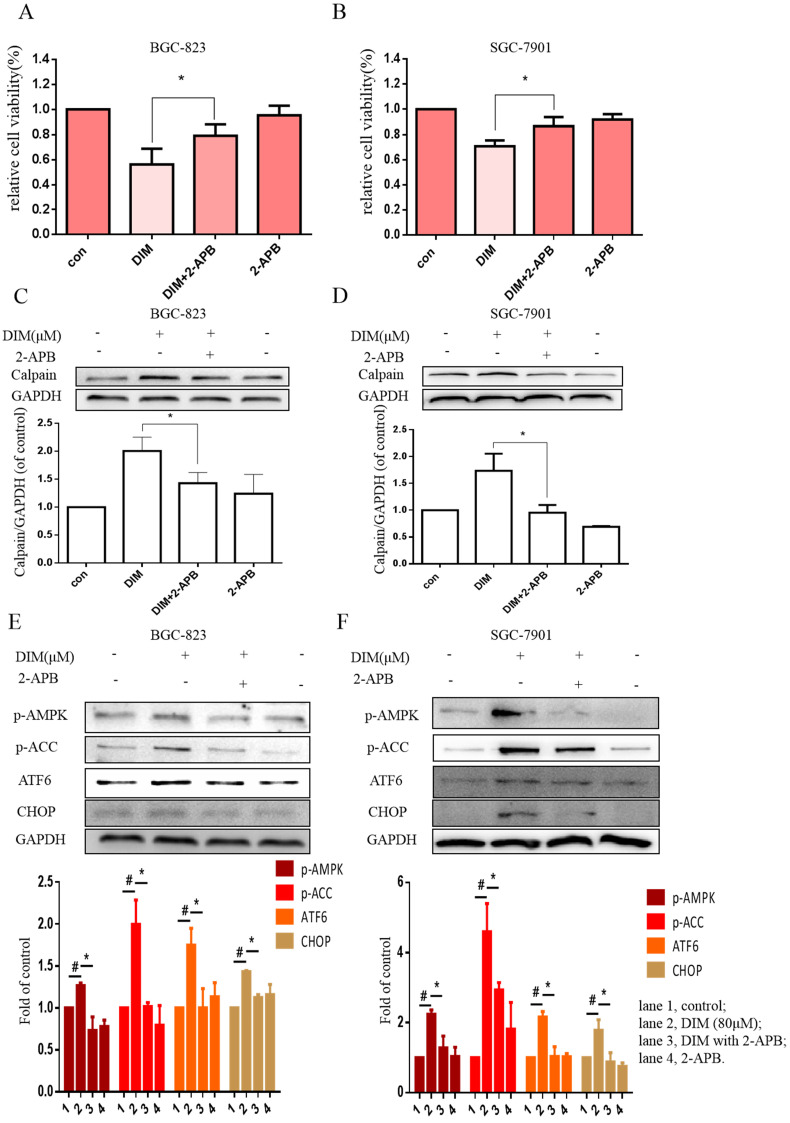

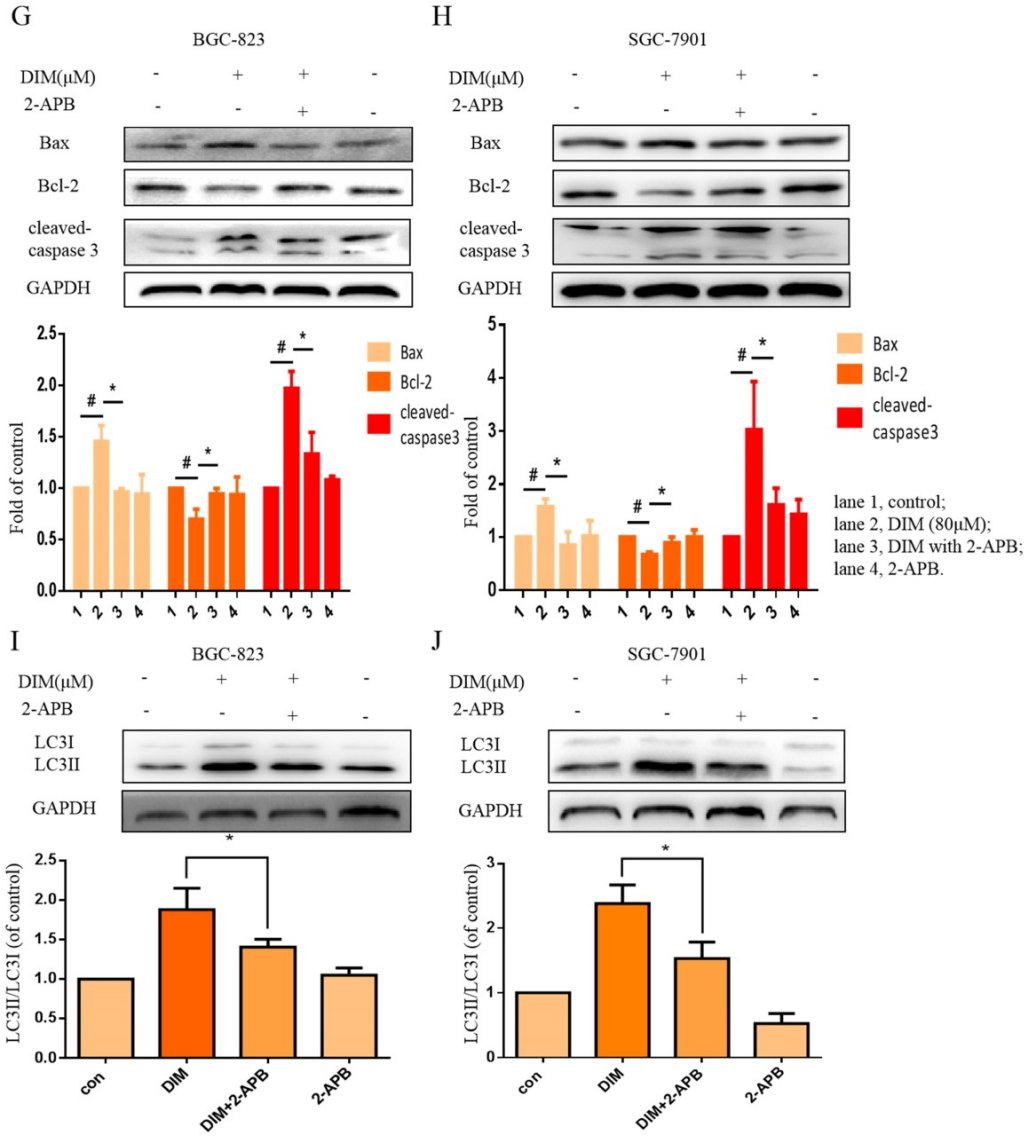
** Effects of SOCE inhibitor 2-APB on DIM-induced cell death and p-AMPK-mediated ER stress in gastric cancer.** (A, B) Cells were treated with DIM (80μM), 2-APB (20μM) for 24h, cell viability were detected by MTT assay. The results are presented as mean±SD and described as column chart *p<0.05 as compared with control group. (C, D, E, F, G, H, I, J) Cells were treated with DIM (80μM), 2-APB (20μM) for 24h, the expression of Calpain, p-AMPK, p-ACC, ATF6, CHOP, Bax, Bcl-2, cleaved-caspase 3, LC3II/LC3I were detected by Western blot. (The data represent mean± SD of three independent experiments (n=3) (*p<0.05 compared with the control group, #p<0.05 compared with the DIM group)).

The effect of SOCE on DIM-induced cells death was further explored by knockdown of a SOCE component STIM1 and pharmacological inhibition. STIM1 siRNA was transfected into BGC-823 gastric cancer cells, the knockdown efficiency was shown in Figure 6E, cells proliferation inhibition was reversed after co-treatment of DIM and STIM1 siRNA (Figure 6F). Furthermore, silencing of STIM1 significantly blocked the up-regulation of p-AMPK, p-ACC, ATF6, CHOP by DIM. In addition, DIM-induced Bax, cleaved-caspase 3, LC3II/LC3I and decreasement of the Bcl-2 was also attenuated (Figure 6G-L). Consistently, 2-APB weakened DIM-induced cells death in BGC-823 and SGC-7901 gastric cancer cells (Figure 7A, B). Moreover, compared to the DIM-only treatment group, the combination treatment of 2-APB and DIM significantly reversed the levels of Calpain, p-AMPK, p-ACC, ATF6 and CHOP (Figure 7C-F), also Bax, cleaved-caspase 3, LC3II/LC3I were significantly decreased and Bcl-2 was increased (Figure 7G-J). Taken together, SOCE may be involved in DIM-induced cells apoptosis and autophagy by p-AMPK mediated ER stress.

## Discussion

The present study examined for the first time that DIM targeting activate STIM1-mediated SOCE and significantly increased the cytoplasmic Ca^2+^ overload, which in turn enhance p-AMPK/p-ACC mediated ER stress to induce apoptosis and autophagy. Knockdown of STIM1 and pharmacological inhibition of SOCE could reverse this effect by reducing intracellular Ca^2+^ accumulation and inhibiting p-AMPK mediated ER stress. These suggested that STIM1-mediated SOCE activation may become a promising therapeutic target to enhance effectiveness of DIM for gastric cancer therapy. In addition, more attention should be paid to combination chemotherapy of anti-cancer drugs targeting SOCE for clinical application. Some phytochemicals exert anti-cancer effect via inhibiting STIM1-mediated SOCE such as curcumin and phemindole 35, 36, by contrast, DIM activate STIM1-meidated SOCE to inhibit gastric cancer cells, the combination of these may reduce the effectiveness of anti-cancer. It should be attentive to recommend the patients with cancer taking anti-cancer drugs acting on STIM1-mediated SOCE with synergistic effect.

Our previous studies have reported that DIM induces cells apoptosis and autophagy to inhibit proliferation in gastric cancer 4 and liver cancer 8. In the present research, we mainly investigate the mechanism in DIM-induced gastric cancer cells death. Given ER stress is one of the main way to regulating apoptosis and autophagy 37, a recent study reported that Chelerythrine induced apoptosis related to ER stress in human renal cell carcinoma 38, we considered whether an relationship existed between ER stress and anti-cancer effect by DIM. Similarly, we observed that DIM activates ER stress and UPR in BGC-823 gastric cancer cells, and the inhibition of ER stress could attenuate cells apoptosis and autophagy. The results initially suggested that ER stress may be involved in DIM induces apoptosis and autophagy.

AMPK, a crucial cellular energy sensor regulating metabolic processes 39, has been reported as a novel tumor suppressor in various cancers 40. Moreover, the activation of p-AMPK was associated with ER stress, which led to cells death 43. Studies have shown that metformin can enhance the anti-cancer effect of dasatinib mainly through p-AMPK mediated ER stress 40. Consecutively, in our study, whether p-AMPK/p-ACC involved in regulating anti-cancer by DIM-mediated ER stress is added. We verified that DIM regulated ER stress through the induction of p-AMPK/p-ACC, following induced cells apoptosis and autophagy. These indicated that the p-AMPK/p-ACC mediated ER stress participated in DIM-induced anti-cancer effect.

Dysregulation of cytoplasmic Ca^2+^ homeostasis involved in many cancer processes such as proliferation, migration and apoptosis 42. We observed sustained Ca^2+^ overload by DIM, BAPTA-AM blocked DIM-induced Ca^2+^ and cells death, suggesting that Ca^2+^ homeostasis impacted the sensitivity of DIM. Furthermore, Ca^2+^ release from ER Ca^2+^ store and SOCE are necessary in maintenance of [Ca^2+^]i homeostasis 43. STIM1 act as Ca^2+^ sensor on the ER membrane and mediate SOCE, which was widely responsible for cell survival and death in various of cancer 36, 44-45. Xia reported that elevated expression of STIM1 promote cells proliferation and metastasis in human gastric cancer cells 33. Yang found that STIM1 mediated SOCE increase cytoplasmic Ca^2+^ levels to result in imatinib resistance in gastrointestinal stromal tumors 46. These suggested that STIM1-mediated SOCE contribute to tumorigenesis in some cancers. In contrast, Liu reported that SOCE enhance cytoplasmic Ca^2+^ overload to inhibit cells growth in glioblastoma 47. In gastric cancer cells, nucleotides activate SOCE, which raise Ca^2+^ levels to exert anti-cancer effect 48. It was demonstrated that STIM1-mediated SOCE may play dual roles in types of cancer under the treatment of different anti-cancer drugs. The in-depth understanding of STIM1 mediated SOCE may be an promising subject to improve effect in cancer therapy. In the present study, we showed that DIM significantly upregulated STIM1 and decrease of ER Ca^2+^ levels, indicating that DIM cause STIM1 mediated SOCE activation and sustained cytoplasmic Ca^2+^ overload in gastric cancer. Moreover, knockdown of STIM1 and pharmacological inhibition of SOCE significantly attenuate cytoplasmic Ca^2+^ overload and reverse DIM-induced cells apoptosis and autophagy, suggesting that STIM1-mediated SOCE involved in DIM induced cells death. In addition, some studies have shown that STIM1-meidiaed SOCE as trigger of signaling pathway activating a serious of downstream signal 45. We also found that knockdown of STIM1 and pharmacological inhibition of SOCE could regulate p-AMPK/ER stress pathway, it suggested that STIM1 as an upstream molecular for DIM induced cells apoptosis and autophagy was associated with p-AMPK mediated ER stress. A report also has been shown that Camptothecin induce autophagy mainly through Ca^2+^ mediates AMPK signaling pathway 49, which are consistent with our results. These suggest that SOCE may be an attractive upstream targets in cancer treatment, which enhance anti-cancer effect of DIM.

## Conclusion

**Figure 8:**
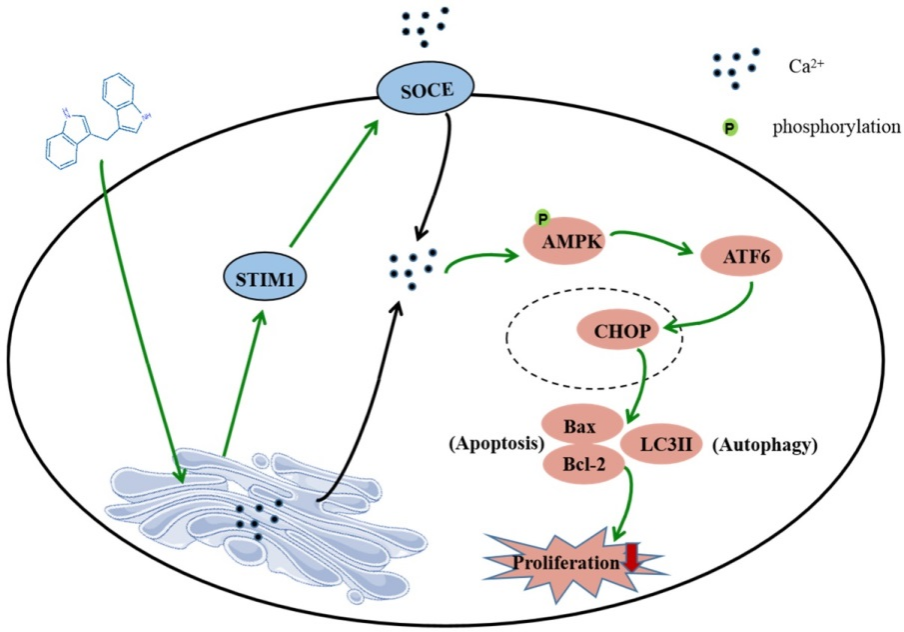
Schematic of the mechanism via which DIM mainly target SOCE to induce BGC-823 gastric cancer cells apoptosis and autophagy through Ca^2+^/AMPK/ER stress signaling pathway. DIM activate STIM1-mediated SOCE, which significantly increase intracellular Ca^2+^ concentration and subsequently leads to the activation of p-AMPK/p-ACC expression and ER stress. In ER stress, DIM mainly activate the ATF6-CHOP signaling pathway. In addition, AMPK signaling pathway is also a mediator of ER stress, which leads to apoptosis and autophagy.

In summary, this study shows that DIM induces apoptosis and autophagy by p-AMPK mediated ER stress in BGC-823 and SGC-7901 gastric cancer cells. The STIM1 mediated SOCE activation also involved in this process, and the knockdown of STIM1 or pharmacological inhibition of SOCE can reversed anti-cancer effect of DIM by inhibiting p-AMPK-mediated ER stress, indicating that STIM1 mediated SOCE might be a critical step for DIM induced cells death (Figure 8). Therefore, our study identified an effective way to activate STIM1-mediated SOCE, which may enhance the sensitivity of targeting Ca^2+^ homeostasis phytochemicals in gastric cancer.
